# Applications of Artificial Intelligence (AI) in Breast Cancer Care Delivery and Education: A Scoping Review

**DOI:** 10.3390/ijerph23050545

**Published:** 2026-04-23

**Authors:** Princella Ntumwine Seripenah, Prudence Ikechukwu, Georgette Oni, Susanna Polotto, William Adeboye, Jo Leonardi-Bee, Chloe Jordan, Joanne Morling, Fatimah Aiyelabegan, Surakshya Dhungana, Heidi Emery, Elisa Martello, James Stewart-Evans, Catrin Evans, Jaspal Taggar, Emma Wilson

**Affiliations:** 1 Centre for Public Health and Epidemiology, School of Medicine, University of Nottingham, Nottingham NG7 2UH, UK; princella.seripenah2@nottingham.ac.uk (P.N.S.); prudenceik198@gmail.com (P.I.); georgette.oni@nhs.net (G.O.); jo.leonardi-bee@nottingham.ac.uk (J.L.-B.); joanne.morling@nottingham.ac.uk (J.M.); fatimahaiyelabegan@gmail.com (F.A.); iamsurakshya@outlook.com (S.D.); heidi.emery@nottingham.ac.uk (H.E.); elisa.martello2@nottingham.ac.uk (E.M.);; 2 Centre for Academic Primary Care, School of Medicine, The University of Nottingham, Nottingham NG7 2UH, UK; jaspal.taggar@nottingham.ac.uk; 3 Cambridge University Hospitals NHS Trust, Hills Road, Cambridge CB2 0QQ, UK; william.adeboye2@nhs.net (W.A.); chloe.jordan10@nhs.net (C.J.); 4 Nottingham Breast Institute, Nottingham University Hospitals NHS Trust, Nottingham NG5 1PB, UK; s.polotto@nhs.net; 5 Faculty of Medicine and Health Science, School of Life Sciences, University of Nottingham, Nottingham NG7 2UH, UK; 6 Centre for Evidence Based Healthcare, School of Medicine, University of Nottingham, Nottingham NG7 2UH, UK; catrin.evans@nottingham.ac.uk

**Keywords:** artificial intelligence, breast cancer, machine learning, post-diagnosis care, scoping review, patient education, recurrence prediction, large language models, survivorship, conversational agents

## Abstract

**Highlights:**

**Public health relevance—How does this work relate to a public health issue?**

- Breast cancer is the most commonly diagnosed cancer among women worldwide, and AI technologies are increasingly being developed to support care after diagnosis. Yet, no comprehensive map exists of how these tools are applied across the post-diagnosis care pathway.
- The rapid growth of generative AI tools such as ChatGPT has created new pathways for patients to independently access cancer-related health information, with uncertain implications for safety and equity.

**Public health significance—Why is this work of significance to public health?**

- This scoping review identifies 54 studies across four post-diagnosis care stages, revealing that 83% of AI applications are provider-focused and concentrated in recurrence prediction, while patient-facing tools and palliative care remain largely unaddressed.
- The evidence base is dominated by retrospective designs from high-income countries, raising concerns about the generalisability and equitable implementation of AI tools across diverse populations and healthcare settings.

**Public health implications—What are the key implications or messages for practitioners, policymakers, and/or researchers in public health?**

- Policymakers and health systems should distinguish between clinically integrated conversational agents and unsupervised generative AI when developing governance frameworks for AI in cancer care.
- Future research should prioritise prospective evaluation of AI tools in real-world clinical workflows, patient experience studies, and the development of AI applications in underserved care stages such as survivorship and palliative care.

**Abstract:**

Artificial intelligence (AI) is increasingly being applied in breast cancer care, yet its use across the post-diagnosis phase remains poorly mapped. This scoping review aimed to identify and categorise AI applications in post-diagnosis breast cancer care, encompassing treatment planning, treatment delivery, follow-up and surveillance, survivorship, and palliative care. Following JBI methodology and PRISMA-ScR reporting guidelines, four databases (MEDLINE, EMBASE, CINAHL, and Web of Science) were searched, identifying 3784 records. After screening and full-text assessment, 54 studies published between 2016 and 2024 were included. Machine learning was the predominant technology (81%), followed by generative AI (7%), conversational agents (6%), traditional natural language processing (4%), and data mining (2%). Follow-up and surveillance were the most represented care stage (48%), driven primarily by recurrence prediction models. Most applications were provider-focused (83%), while patient-facing tools accounted for 17% of studies and relied on either conversational agents or generative AI. No studies addressed palliative care. The evidence base was predominantly retrospective (70%) and concentrated in high-income countries (74%). Future research should prioritise prospective evaluation in clinical workflows, address unsupervised patient use of generative AI, and ensure equitable development across diverse populations and care settings.

## 1. Introduction

Breast cancer remains the most commonly diagnosed cancer among women worldwide, with an estimated 2.3 million new cases in 2020 and approximately 670,000 deaths in 2022, accounting for 11.7% of all cancer diagnoses globally [1,2]. Management often involves multimodal interventions involving surgery, radiotherapy, chemotherapy, hormone therapy, and biological therapies tailored to tumour subtype, genetic profile, and patient preferences [3,4]. This means that no single treatment fits every breast cancer patient, and care must therefore be individualised to each patient’s clinical profile, disease characteristics, and personal circumstances. Such complexity in treatment demands adaptive, data-driven approaches to personalise care and reduce the risk of over- or under-treatment. With increasing breast cancer survival rates, more patients are living longer with treatment side effects, fear of recurrence, and ongoing care needs [5,6].

Traditional digital tools in healthcare often rely on static programming; however, artificial intelligence (AI) introduces a fundamental shift by enabling systems to learn from data, adapt to new information, and mimic aspects of human reasoning. These capabilities are driven by the integration of large datasets, advanced algorithms, and powerful computing infrastructure [7,8]. In cancer care, machine learning (ML) [7,9,10], traditional natural language processing (NLP) [11,12], and data mining [13] are increasingly applied to interpret complex clinical data, support decision-making, and personalise treatment strategies. ML identifies patterns in large datasets without explicit programming, while traditional NLP enables computers to process and generate human language, capabilities that make these technologies particularly suited to complex clinical environments. These tools can help uncover patterns across large populations, predict outcomes at the individual level, and make care more responsive and efficient.

Effective communication is critical in healthcare, especially when conveying complex medical information to patients. Studies indicate that communication in a patient’s native language by relatable healthcare professionals fosters improved understanding [14]. However, this is not always feasible, particularly in diverse, multicultural settings. AI has been applied in a range of clinical contexts, from supporting treatment planning and clinical decision-making to monitoring patients during and after treatment. AI-enabled tools such as chatbots, personalised treatment planners, and traditional NLP-powered education platforms have emerged as promising approaches to bridge these gaps in breast cancer care. For example, AI chatbots like ChatGPT have been shown to provide accurate, comprehensible, and reliable responses to breast cancer-related questions, outperforming traditional search engines in depth and quality [15,16,17,18], and have demonstrated potential in reducing misinformation by delivering structured, fact-based information [19]. NLP systems can simplify clinical language to make medical content more accessible across diverse populations [11,12], while data mining and ML models can detect behavioural or clinical patterns that inform targeted interventions [13,20,21].

Despite these promising developments, important questions remain about the reliability and limitations of AI in clinical practice. Haug and Drazen caution that, despite impressive technical performance, AI and ML systems in medicine face significant challenges, including risk of bias, lack of transparency, and uncertain generalisability across diverse populations [20]. Similarly, concerns have been raised about whether current tools, many developed and validated in high-resource, largely homogeneous datasets, can be reliably deployed across multicultural and low-resource settings without exacerbating existing health inequities [8,21]. Proponents counter that thoughtfully implemented AI, grounded in rigorous validation frameworks, holds genuine potential to democratise access to high-quality health information and reduce the burden on overstretched healthcare systems [7,13]. These tensions highlight the need for robust, evidence-based synthesis of how AI performs in patient-facing breast cancer care, rather than relying solely on technical benchmarks.

**Figure 1:**
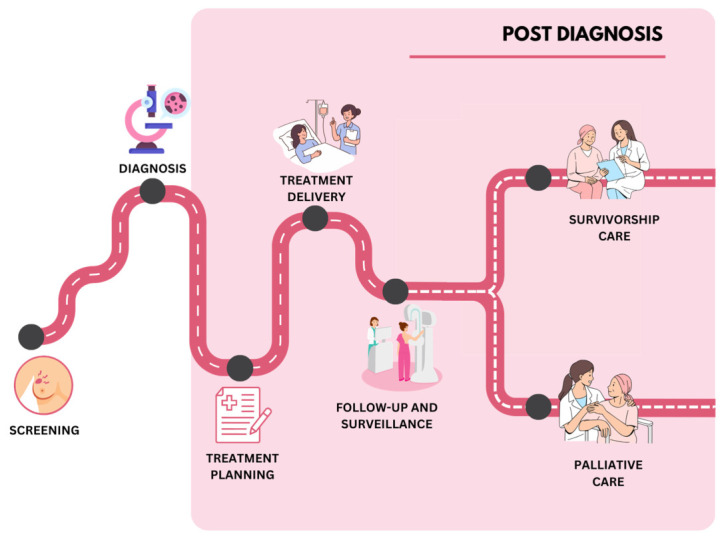
The breast cancer care journey highlighting the post-diagnosis phase of care. Image created by the authors using Canva (Canva Pty Ltd., Sydney, Australia). https://www.canva.in/ (accessed on 20 October 2025).

A preliminary search identified prior scoping reviews examining AI applications in breast cancer; however, these primarily focused on pre-diagnosis and diagnostic stages such as screening and imaging [22,23], or examined the full care journey without comprehensively addressing the post-diagnosis phase [24]. To our knowledge, no review has systematically examined the application of AI technologies across post-diagnosis breast cancer care. This scoping review aimed to map and synthesise existing evidence on the application of AI technologies in post-diagnosis breast cancer care. The post-diagnosis care stages examined in this review are illustrated in Figure 1.

## 2. Materials and Methods

The review was conducted in accordance with the JBI methodology for scoping reviews [25] and reported in line with the Preferred Reporting Items for Systematic Reviews and Meta-Analyses Extension for Scoping Reviews (PRISMA-ScR) [26] (Supplementary File S4). The study protocol was registered with the Open Science Framework [27]. The primary research question for this review was as follows: “What is known from existing literature about the application of AI technologies in post-diagnosis breast cancer care?”

### 2.1. Eligibility Criteria

#### 2.1.1. Participants

The initial protocol specified solely the inclusion of individuals diagnosed with breast cancer as the population of interest. However, a substantial portion of the literature focuses on healthcare providers who implement or engage with patient-facing AI technologies. Therefore, the population was amended to include both breast cancer patients and healthcare providers to ensure a comprehensive synthesis of relevant evidence.

Studies were therefore eligible if they involved the following:

- Patients with a confirmed diagnosis of breast cancer, regardless of age, gender, or socioeconomic status, who are currently receiving or have previously received treatment;
- Healthcare providers who use, deliver, or implement AI technologies in the post-diagnosis phase of breast cancer care.

Studies involving individuals with other cancer types or unrelated medical conditions were excluded.

#### 2.1.2. Concept

For the purpose of this review, AI refers to the capability of algorithms integrated into systems and tools to learn from data so that they can perform automated tasks without explicit programming of every step by a human [28]. In healthcare, AI encompasses a range of technologies, including, but not limited to, ML algorithms, deep learning, NLP, conversational agents (including chatbots), clinical decision support systems, and computer vision or sensor-based tools [29]. These may be either operational (already in use in clinical or supportive care settings) or conceptual, still under development but described with potential applications for patient care.

Eligible studies must focus on the post-diagnosis phase of care, which includes treatment, follow-up, and survivorship. AI applications may be used alongside various treatment modalities such as surgery, chemotherapy, radiotherapy, or hormonal therapy. Studies focused exclusively on initial screening or diagnostic accuracy, prior to a confirmed breast cancer diagnosis, were excluded.

This review encompassed both clinician-facing AI applications, including clinical decision support, outcome prediction, treatment planning, and workflow optimisation, and patient-facing applications [29]. For the purpose of this review, patient-facing education was defined as the provision of information relating to diagnosis, treatment options, side effects, lifestyle adjustments, and prognosis. Patient-facing management refers to the ongoing coordination of care, including monitoring symptoms, supporting treatment adherence, facilitating decision-making, and enhancing communication between patients and providers.

#### 2.1.3. Context

This review included studies situated in urban and suburban healthcare settings, where access to structured breast cancer care and technological infrastructure is more readily available. While acknowledging the influence of cultural, socioeconomic, and geographic diversity on healthcare delivery, the focus is on contexts in which AI tools are implemented within formal healthcare systems serving these populations. Studies conducted in rural or underserved settings, where access to healthcare and digital technologies may be limited, were excluded from the scope of this review [30]. These were excluded to minimise contextual heterogeneity related to healthcare infrastructure. However, future scoping reviews examining AI applications specifically in such settings would be a valuable and necessary complement to this work.

#### 2.1.4. Types of Sources

Studies were included if they reported primary research investigating the use of AI in post-diagnosis breast cancer care. Both peer-reviewed journal articles and conference abstracts were eligible for inclusion, provided sufficient methodological and outcome data could be extracted. Systematic reviews, meta-analyses, and other secondary research were excluded from the mapping and synthesis; however, their reference lists were hand-searched to identify any primary studies that may have been missed during the initial database search.

### 2.2. Search Strategy

The search strategy aimed to identify both published and unpublished studies using a three-step approach. An initial limited search of MEDLINE and CINAHL via the EBSCOhost platform was conducted to identify relevant articles. Text words in the titles and abstracts, along with index terms used to describe the articles, informed the development of a comprehensive search strategy. This strategy was then adapted and applied to MEDLINE (via Ovid), EMBASE (via Ovid), CINAHL (via EBSCOhost), and Web of Science. Given the rapid evolution of artificial intelligence technologies in healthcare, the search was limited to studies published between January 2013 and 10 December 2024 to ensure relevance to contemporary developments and applications. The full search strategy for all databases is presented in Supplementary File S1.

The reference lists of all included articles and previous reviews were screened to identify additional sources. Unpublished studies and grey literature were searched through sources such as conference abstracts and proceedings in Web of Science Conference Proceedings. No language or geographic restrictions were applied.

### 2.3. Selection of Sources of Evidence

Following the database searches, all identified records were collated and uploaded into Rayyan (Rayyan Systems Inc., Cambridge, MA, USA; version accessed December 2024) for deduplication. Titles and abstracts were screened against eligibility criteria by two independent reviewers (PS and PI/FA). Full-text articles of studies potentially eligible for inclusion were retrieved and assessed independently by the same reviewers (PS and PI/FA), with discrepancies resolved through discussion.

### 2.4. Data Charting Process

A tailored data extraction form was piloted using a small subset of studies and refined where necessary. All included studies underwent data extraction by two independent reviewers (PS and PI), using the developed data extraction tool (see Supplementary File S2), with disagreements resolved through consensus. The following details were extracted: article details (author, publication year, country), study design, population, study aim, type of AI technology, role or application of AI technology, and post-diagnosis care stage. Demographic characteristics (e.g., gender and age) were inconsistently reported across the included studies; therefore, these data were not extracted.

### 2.5. Data Analysis and Presentation

Data from the included studies were presented in a table and grouped based on the type of AI technology used, role of AI, population focus of AI tool, year of publication, country, and study design used. A narrative summary was generated for each group, describing the role of the AI technology. Summary statistics are reported where relevant as frequencies with percentages.

AI technologies were classified into five categories based on their primary functional characteristics: machine learning (including classical algorithms, deep learning, and neural networks), generative AI (large language model-based tools), conversational agents (rule-based or scripted chatbot systems), traditional NLP, and data mining. In the case where a study employed multiple techniques, it was classified according to the predominant technology described.

## 3. Results

### 3.1. Source of Evidence Inclusion

**Figure 2:**
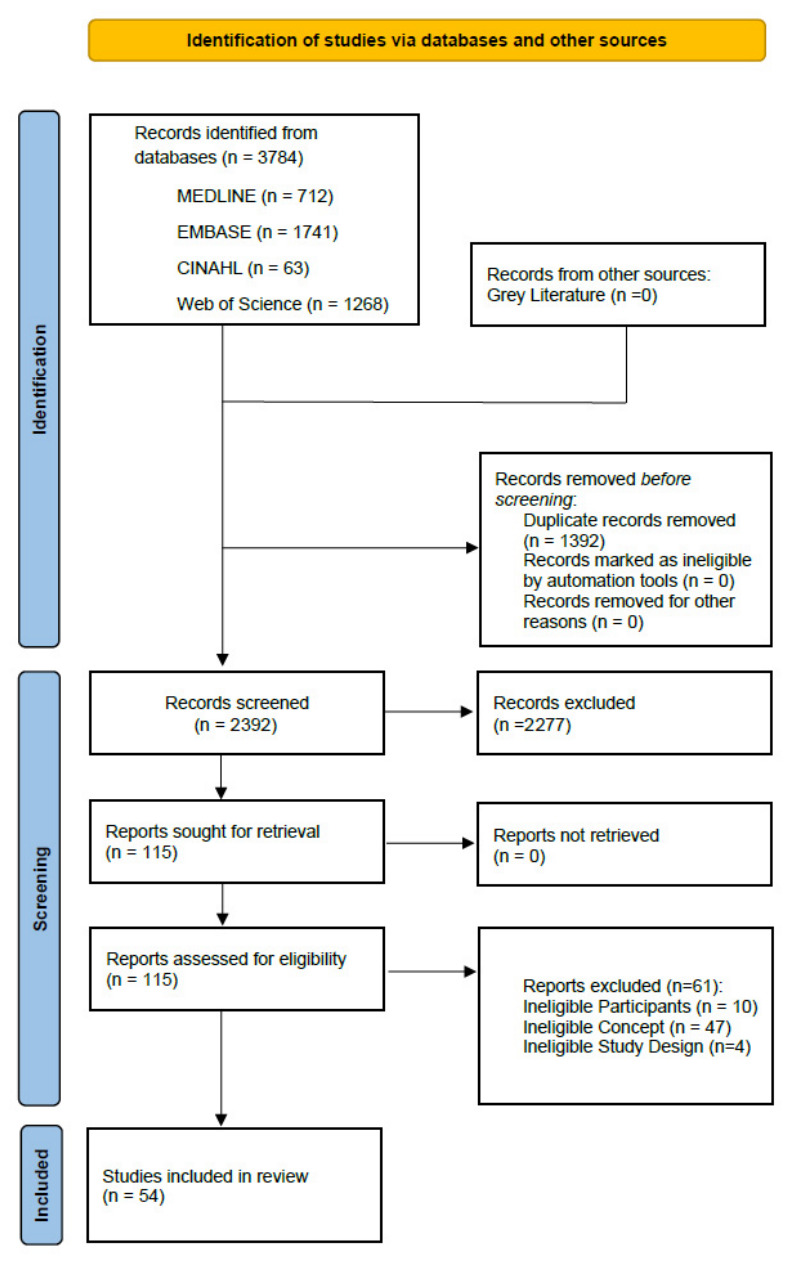
PRISMA flow diagram showing the identification, screening, eligibility, and inclusion of studies examining artificial intelligence applications for patient education and care management in the post-diagnosis phase of breast cancer care.

The database searches identified 3784 records across MEDLINE, EMBASE, CINAHL, and Web of Science. After removing 1392 duplicates, 2392 records were screened by title and abstract, of which 2277 were excluded. The remaining 115 full-text articles were assessed for eligibility. Of these, 61 studies were excluded: 47 for ineligible concept (e.g., AI applied exclusively to screening or diagnosis rather than post-diagnosis care), 10 for ineligible participants (e.g., populations without a confirmed breast cancer diagnosis), and 4 for ineligible study design (3 systematic reviews and 1 study protocol, neither of which constituted primary research with extractable data). A total of 54 studies met the inclusion criteria and were included in the review (Figure 2).

### 3.2. Characteristics of Included Studies

The summary of all 54 included studies is reported in Supplementary File S3. Studies were published between 2016 and 2024, with a marked increase over time: 87% (*n* = 47) were published from 2020 onwards, and over half (52%, *n* = 28) were from 2023 onwards, with 2024 alone accounting for 17 studies (31%). This trajectory reflects the rapid growth of AI research in post-diagnosis breast cancer care during this period.

Geographically, the United States was the most represented country (*n* = 15, including two multi-country studies), followed by the Netherlands and China (*n* = 5 each), Italy (*n* = 4), and India and South Korea (*n* = 3 each). Collectively, European countries contributed the largest share (*n* = 19, 35%), followed by countries in Asia and the Middle East (*n* = 18, 33%) and North America (*n* = 15, 28%). One study did not state its country of origin.

The majority of included studies were published as peer-reviewed journal articles (*n* = 38, 70%), with the remaining 16 (30%) reported as conference abstracts. The predominant study design was retrospective cohort (*n* = 38, 70%), followed by cross-sectional studies (*n* = 6, 11%) and longitudinal studies (*n* = 4, 7%). The remaining six studies comprised a randomised controlled trial, a prospective cohort, a content analysis, a pilot study, a case study, and a comparative observational study.

Machine learning was the most frequently identified AI technology, encompassing 44 studies (81%), including classical algorithms, deep learning architectures, and neural networks. Generative AI, specifically ChatGPT-based tools, was employed in four studies (7%). Three studies (6%) used non-generative conversational agents, that is, rule-based or scripted chatbot systems without LLM capabilities. Traditional NLP was reported in two studies (4%), and data mining in one study (2%).

In terms of implementation focus, a large majority of studies (*n* = 45, 83%) described provider-focused tools supporting tasks such as clinical decision-making, outcome prediction, and workflow optimisation. Nine studies (17%) described patient-focused applications, all of which employed either conversational agents (*n* = 3), generative AI (*n* = 4), or machine learning applied to patient-reported outcomes (*n* = 2).

### 3.3. Review Findings

**Figure 3:**
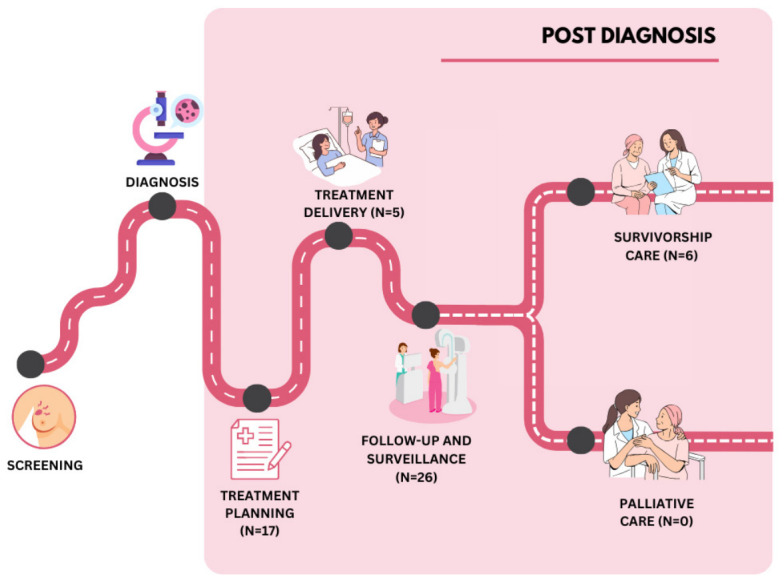
Number of studies reporting the use of AI mapped to stages of the post-diagnosis breast cancer care journey. Image created by the authors using Canva (Canva Pty Ltd., Sydney, Australia).

Studies were categorised according to the stage of the post-diagnosis breast cancer care journey targeted by the AI application: treatment planning, treatment delivery, follow-up and surveillance, and survivorship care (Figure 3). No studies were identified that addressed palliative care.

The largest concentration of AI applications was in follow-up and surveillance, which accounted for nearly half of all included studies (*n* = 26, 48%). Treatment planning was the second most represented stage (*n* = 17, 31%), followed by survivorship care (*n* = 6, 11%) and treatment delivery (*n* = 5, 9%).

#### 3.3.1. AI for Treatment Planning

Seventeen studies [31,32,33,34,35,36,37,38,39,40,41,42,43,44,45,46,47] applied AI to support clinical decisions during the treatment planning stage. Five studies developed models to predict long-term survival based on demographic, clinical, and molecular features [31,39,41,43,47], using approaches ranging from traditional machine learning classifiers to deep learning architectures and automated mitosis detection pipelines. One study specifically predicted recurrence risk scores to guide adjuvant chemotherapy decisions in early-stage disease [35].

Four studies reported the use of AI for workflow optimisation [32,34,37,46]. These tools aimed to improve efficiency and consistency by, for example, forecasting inpatient length of stay after mastectomy [37], identifying treatment patterns to improve planning efficiency [46], extracting treatment parameters from clinical letters [34], and increasing guideline-compliant genomic testing [32].

Three studies applied AI directly to treatment planning processes, including automated radiotherapy plan generation [33], quality prediction of whole-breast radiotherapy plans [38], and development of a personalised follow-up care algorithm for breast cancer survivors transitioning from specialist to primary care [45].

Two studies addressed disease progression risk prediction in the treatment planning context, associating mitotic activity with gene-expression risk categories [42] and predicting case complexity to support multidisciplinary tumour board decision-making [44].

Two studies were patient-facing. One used chatbot technology to educate patients prior to breast biopsy [36], while the other employed machine learning to predict individual patient-reported outcomes to support shared decision-making about mastectomy and reconstruction [40].

The dominant AI technology in treatment planning was machine learning (*n* = 15), with one study using a conversational agent [36] and one using data mining [46].

#### 3.3.2. AI for Treatment Delivery

Five studies [48,49,50,51,52] explored the use of AI during active treatment. Two focused on predicting adverse events: one developed neural network models to predict patient-reported adverse events during radiotherapy using electronic health record data [48], while the other assessed machine learning for individualised risk prediction of mastectomy skin flap necrosis [49].

One study applied a gene-mutation-based algorithm to predict treatment response in triple-negative and other breast cancer subtypes [50].

Two studies were patient-facing, both using chatbot technology to support patients receiving active treatment. One deployed an automated chatbot to collect patient-reported outcomes during radiotherapy [51], and the other conducted a randomised controlled trial comparing a nurse-led intervention with a chatbot-based empowerment programme for managing chemotherapy side effects [52].

Machine learning was used in three studies [48,49,50], and conversational agents were used in two [51,52].

#### 3.3.3. AI for Patient Follow-Up and Surveillance

Twenty-six studies [53,54,55,56,57,58,59,60,61,62,63,64,65,66,67,68,69,70,71,72,73,74,75,76,77,78] applied AI within the context of follow-up and surveillance after primary treatment, making this the most represented care stage.

Eighteen studies focused specifically on recurrence prediction [53,54,55,56,58,59,60,62,63,64,65,67,69,72,73,74,75,77]. These models were developed to estimate the likelihood of local, regional, or distant recurrence using a range of data inputs, including clinicopathological variables, imaging features, genomic signatures, electronic health records, and histopathological slides. Notable among these were three related studies validating the PreciseDx Breast AI-enabled digital test for predicting recurrence within six years: an initial evaluation on a MammaPrint low-risk cohort [58], external validation on an independent cohort [59], and clinical validation using diagnostic biopsy specimens [60]. One study employed traditional NLP to extract recurrence timelines from unstructured clinical notes [53].

Five studies addressed disease progression risk prediction during follow-up [57,61,68,71,76], developing models for longitudinal monitoring of disease progression in metastatic breast cancer [57], invasive disease event prediction using deep reinforcement learning [61], bone lesion segmentation for metastatic disease [68], ocular metastasis prediction [71], and estimation of progression-free survival [76].

Two studies predicted survival outcomes: one applied machine learning to standardise breast cancer grading and develop multivariate risk models [70], and the other predicted mortality integrating clinical and lifestyle factors [78].

One study predicted persistent post-surgery pain using preoperative cold pain sensitivity biomarkers [66], representing the only adverse event prediction model in this care stage.

Machine learning was the predominant AI technology across all 26 studies in this stage (n = 24), followed by two studies using traditional NLP [53,76]. All studies in this category were provider-focused.

#### 3.3.4. AI for Survivorship Care

Six studies [16,17,18,19,79,80] explored AI applications in the survivorship phase. Four studies evaluated generative AI, specifically ChatGPT, as a resource for patient education, assessing its accuracy and utility in responding to patient queries about breast cancer care, reconstruction, and metastatic disease [16,17,18,19]. One study used machine learning to predict individual patient-reported outcomes at a two-year follow-up after cancer-related mastectomy and breast reconstruction, supporting personalised survivorship planning [80].

In contrast, one study took a provider-focused approach, developing a deep learning model to predict cardiovascular disease risk among long-term breast cancer survivors [79].

Survivorship care was the only stage where generative AI constituted the dominant technology (4 of 6 studies), reflecting the recent emergence of large language models in patient-facing cancer care applications. It was also the most patient-focused stage, with five of the six studies oriented toward patient education and support.

#### 3.3.5. AI for Palliative Care

No studies were identified that applied AI to palliative care for breast cancer patients, representing a notable gap in the current literature.

## 4. Discussion

### 4.1. Summary of Evidence

This scoping review mapped the application of AI technologies across the post-diagnosis care pathway in breast cancer. Of the 54 included studies, most were situated within the follow-up and surveillance stage, with fewer studies focused on treatment planning, delivery, or survivorship care. The predominant AI functions identified were recurrence prediction, survival forecasting, and workflow optimisation. Despite increasing interest in AI-enhanced care, no studies were identified that directly addressed palliative or end-of-life disease management.

#### 4.1.1. AI in Prognostic Modelling and Clinical Workflows

Most included studies described provider-focused tools (83%), particularly those leveraging ML for recurrence prediction, survival forecasting, and treatment pathway optimisation. Machine learning was the most frequently applied AI technology (81% of studies), likely due to its capacity to analyse large, complex datasets, including medical imaging, genomic profiles, and electronic health records [9]. Within the machine learning category, studies employed a range of approaches, including classical algorithms (such as logistic regression and random forests), deep learning architectures (such as convolutional neural networks), and ensemble methods. For clinical oncologists, such models may offer decision support in areas such as adjuvant therapy planning, margin assessment, and recurrence risk stratification. However, the reviewed literature offered limited detail on how these predictions are operationalised within multidisciplinary care or how outputs are communicated to patients. Across the included studies, most AI applications reflect early-stage algorithm development based on retrospective datasets with internal validation only. A smaller subset described external or independent cohort validation, and very few studies reflected prospective evaluation or real-world clinical deployment. While these tools show promise in augmenting clinical judgment, few studies have explored their implementation in real-world workflows or their interpretability for clinicians.

Within this body of work, traditional NLP was used in two studies to extract clinically meaningful information from unstructured electronic health records, including recurrence timelines [53] and progression-free survival estimates [76]. While technically distinct from machine learning classification models, these approaches share a provider-facing orientation and highlight the potential of AI to unlock value from routinely collected clinical text. Data mining was used in one study to discover treatment patterns from structured datasets [46]. These less frequently reported technologies may be underrepresented in the literature relative to their potential utility.

#### 4.1.2. Patient-Facing AI: Conversational Agents and Generative AI

Nine studies (17%) focused on patient-facing AI applications. These employed two functionally distinct categories of technology: non-generative conversational agents and generative AI. Three studies used conversational agents to support patient education prior to biopsy [36], collect patient-reported outcomes during radiotherapy [51], and empower women to manage chemotherapy side effects [52]. These tools operated within defined conversational pathways and were designed for specific clinical tasks. Recent qualitative research highlights the broader potential of AI-powered chatbots and virtual assistants to enhance patient engagement, emotional support, and treatment adherence in oncology [81]. However, the conversational agents identified in this review were evaluated in experimental or simulated settings, and none were examined during perioperative or post-surgical recovery, an area where structured guidance and psychosocial support could have a practical impact.

Four studies evaluated generative AI, specifically ChatGPT, as a resource for patient education during survivorship [16,17,18,19]. These studies assessed the accuracy, completeness, and readability of LLM-generated responses to patient queries about breast cancer care, reconstruction, and metastatic disease. Unlike the scripted conversational agents described above, generative AI tools produce novel responses derived from broad training data and are not constrained by predefined conversational pathways. While their potential to democratise access to health information is recognised, the evidence base for their clinical integration remains limited.

The distinction between these two technology types carries important implications. Conversational agents, by operating within defined parameters, offer greater predictability and safety but limited flexibility. Generative AI offers richer, more adaptive interaction but introduces risks related to factual accuracy, hallucination, and inconsistency [82,83]. Importantly, individualised questions, such as interpreting risk scores or evaluating treatment trade-offs, require clinical judgment and contextual understanding that extends beyond the current capabilities of either technology and warrant human oversight.

This distinction is especially pertinent considering the growing availability of open-access large language models. Unlike clinically integrated conversational agents, these models can be accessed independently by patients and are not constrained by safety guardrails or scope-of-practice boundaries. None of the included studies examined this emerging phenomenon, representing a gap in the literature. Unsupervised use of general-purpose LLMs for cancer-related guidance raises concerns around misinformation, misinterpretation, and erosion of the therapeutic relationship [84].

These limitations point to the need for multimodal and clinically integrated approaches, where conversational AI tools, whether rule-based or generative, are embedded within care pathways and supported by clinician oversight. The governance of AI tools in cancer care extends beyond informational accuracy. Hallucination in generative AI systems poses particular risks in oncological contexts where patients may act on incorrect AI-generated guidance without clinician oversight [82,83]. Algorithmic bias in models trained on non-representative datasets may further exacerbate health inequities across demographic groups [85,86]. Data privacy, informed patient consent, and clinical accountability for AI-generated outputs must also be carefully considered [28], and deployment of patient-facing AI tools should be supported by robust governance processes and compliance with emerging regulatory frameworks. Future studies should assess not only the informational accuracy of these tools but also their role in complementing human care, particularly in emotionally complex or high-stakes clinical decisions.

#### 4.1.3. Growth in Interest and Versatility of AI for Breast Cancer Care Delivery

This review identified a steady growth in AI applications for breast cancer care, with the number of studies increasing from one in 2016 to 17 in 2024. The increase was particularly pronounced in recent years, with 87% of included studies (*n* = 47) published from 2020 onwards and over half (52%, *n* = 28) from 2023 onwards. This trend likely reflects a broader acceleration in AI applications across various fields during the same period, driven by the widespread availability of open-source ML frameworks and, more recently, LLMs [87].

ML was applied across all four care stages but was most concentrated in follow-up and surveillance, where 24 of 26 studies used machine learning for recurrence prediction, disease progression monitoring, or survival forecasting. Generative AI was exclusively found in the survivorship care stage, where all four LLM-based studies evaluated ChatGPT for patient education. Conversational agents spanned treatment planning and treatment delivery, reflecting their utility in structured patient communication tasks at specific points in the care journey.

Despite these advancements, few studies described how AI-generated insights were integrated into patient education. In most cases, the potential for AI to support shared decision-making or improve patient understanding was discussed in theoretical terms rather than demonstrated in practice. This suggests a gap between the development of predictive models and their use in patient-facing communication.

Several studies also noted implementation challenges, including the need for ongoing model retraining, clinician training, and alignment with data protection standards. There is a risk that models developed in specific populations may not perform consistently across diverse groups [85], underscoring the need for external validation and equitable design.

#### 4.1.4. Disparities in the Use of AI for Breast Cancer Care

In line with global patterns in digital health research, most studies originated from high-income countries, particularly the United States (*n* = 15), the Netherlands (*n* = 5), and Western European nations. Of the 54 included studies, 40 (74%) were conducted in high-income settings. This concentration reflects disparities in infrastructure, funding, and data availability that may limit generalisability. Although many AI technologies are theoretically accessible through open-source platforms, implementation depends on health system readiness, digital literacy, and local workflows.

Although the included studies did not provide extensive data on disparities, it is reasonable to anticipate that age, educational background, and ethnicity could influence how patients interact with and benefit from these tools [86,88,89]. Furthermore, AI tools developed and validated predominantly on patient populations from high-income, Western healthcare settings may not adequately reflect the clinical presentations, tumour biology, and treatment patterns of patients in other regions. This geographic concentration in model development raises legitimate concerns about algorithmic bias, as models trained on non-representative datasets may perform less reliably when applied to demographically distinct populations [86,89]. For patient-facing applications such as survivorship support and education tools, cultural adaptability presents an additional challenge, as health beliefs, communication preferences, and care expectations vary considerably across populations and may not be accommodated by tools designed within a single cultural context.

Moreover, our findings reveal a predominant focus on care providers as primary users of AI systems (83% of studies were provider-focused). While the importance of enhancing the capabilities of all healthcare professionals involved in breast cancer care through AI is evident, a more inclusive approach, considering the perspectives and experiences of patients, is essential for fostering patient-centred care. It is also crucial to remember that these tools do not exist in isolation; the social aspects and the patient–clinician relationship are key factors that need to be considered. AI technologies should complement, rather than replace, the human element in healthcare [29], ensuring that the implementation of these tools enhances, rather than hinders, the patient experience and care outcomes.

### 4.2. Study Limitations

This review has several limitations. Firstly, the review excluded opinion pieces and non-empirical literature, which may have limited broader perspectives on AI integration in breast cancer care. While the review aimed to map AI applications for patient education and care management in the post-diagnosis phase, many included studies did not fully address these concepts. The majority focused on predictive modelling or workflow support, with fewer exploring direct patient-facing tools or educational interventions. As a result, important elements such as shared decision-making, self-management support, and communication were underrepresented. Additionally, the search cut-off of December 2024 means that studies published in 2025 and beyond are not captured in this review. Given the rapid pace of development in generative AI and large language models since that date, future reviews may identify a substantially different landscape of patient-facing AI applications in breast cancer care.

The high proportion of conference abstracts (30% of included studies) limited the depth of data available for extraction, as these sources typically provide less methodological detail than full journal articles. Additionally, the predominance of retrospective cohort designs (70%) means that the evidence base largely reflects model development and validation rather than a prospective evaluation of AI tools in clinical practice.

Finally, few studies examined contextual or implementation factors such as clinician readiness, training needs, or integration into existing clinical workflows. Qualitative insights into patient and provider experiences with AI were also largely absent, restricting the review’s ability to capture relational, ethical, or practical dimensions of AI use in breast cancer care. Future systematic reviews applying more restrictive eligibility criteria or formal quality appraisal tools could enable a more rigorous technical evaluation of AI models in this field.

### 4.3. Comparison with Existing Literature

A previous scoping review examined artificial intelligence applications across the entire breast cancer care pathway, with most studies focused on pre-diagnosis and diagnostic stages, including screening, imaging, and histopathology [24]. While some overlap existed with our included studies, the present review differs in scope and emphasis. We specifically excluded early detection and diagnostic imaging studies to focus on how AI supports patient education, communication, and care management after diagnosis. This review organises the post-diagnosis phase into five stages (treatment planning, treatment delivery, follow-up and surveillance, survivorship care, and palliative care), thus providing a novel synthesis of how AI contributes to patient-centred care beyond the point of diagnosis.

### 4.4. Implications for Future Research and Practice

Future research should explore AI adaptation and scalability in low- and middle-income countries, considering infrastructure, data availability, and socioeconomic factors. This would help ensure that AI benefits are globally accessible and applicable. There is also a critical need for studies centred on breast cancer patients’ experiences and preferences to provide a comprehensive understanding of human factors influencing AI adoption in patient care.

The functional distinction between conversational agents and generative AI identified in this review suggests different research priorities for each. For conversational agents, further work is needed to evaluate their effectiveness within structured care pathways, including perioperative support and self-management during active treatment. For generative AI, research should urgently address the implications of unsupervised patient access to general-purpose LLMs, including studies examining how patients use these tools, what decisions they inform, and what harms may result.

Future research should also focus on the cost-effectiveness of AI technologies, particularly in publicly financed healthcare models, where budget constraints are a concern. AI’s potential to optimise clinical workflows and improve diagnostic and therapeutic precision could lead to more efficient use of resources, translating into lower costs and increased clinician availability. This could be particularly beneficial in publicly funded health systems, where reducing wait times and maximising resource allocation are ongoing challenges.

However, economic benefits must not overshadow the need for safety. AI technologies must be carefully evaluated within specific clinical contexts to ensure they enhance, rather than compromise, patient outcomes. This necessitates cohort studies and higher levels of evidence to assess their real-world impact. As AI evolves, balancing efficiency with safety will be crucial for broader adoption. Ethical implications and integration into clinical practice must be continually evaluated [28], with a focus on equitable benefit distribution among diverse populations and healthcare settings. Integrating qualitative methodologies into research designs will be instrumental in capturing the complex narratives and perceptions surrounding AI in breast cancer care.

## 5. Conclusions

This scoping review identified 54 studies applying AI across four stages of the post-diagnosis breast cancer care journey. ML dominated the evidence base, primarily in provider-focused applications for recurrence prediction and clinical workflow support. Patient-facing applications accounted for few studies and relied on two functionally distinct technologies: non-generative conversational agents for structured patient support during treatment and generative AI (ChatGPT) for patient education during survivorship. Follow-up and surveillance were the most represented care stage, while palliative care remained entirely unaddressed.

The rapid growth in publications signals strong and accelerating research interest, but the evidence base remains predominantly retrospective and concentrated in high-income settings. Future research should prioritise prospective evaluation of AI tools in real-world clinical workflows, address the implications of unsupervised patient access to generative AI, and ensure equitable development and validation across diverse populations and healthcare contexts. As AI continues to reshape breast cancer care, its integration must be guided by the experiences of patients, insights from healthcare providers, and a commitment to patient-centred, evidence-based practice.

## Data Availability

The original contributions presented in this study are included in the article/Supplementary Materials. Further inquiries can be directed to the corresponding author.

## Supplementary Materials

The following supporting information can be downloaded at: https://www.mdpi.com/article/10.3390/ijerph23050545/s1, Supplementary File S1: Full electronic search strategies presented exactly as run in each database; Supplementary File S2: Blank data extraction instrument; Supplementary File S3: Summary table of data extracted from included studies; Supplementary File S4: A completed PRISMA-ScR checklist.

## Abbreviations

The following abbreviations are used in this manuscript:

AI	Artificial Intelligence
ML	Machine Learning
NLP	Natural Language Processing
LLM	Large Language Model

## References

[1] Sung H., Ferlay J., Siegel R.L., Laversanne M., Soerjomataram I., Jemal A., Bray F. (2021). Global Cancer Statistics 2020: GLOBOCAN Estimates of Incidence and Mortality Worldwide for 36 Cancers in 185 Countries. CA Cancer J. Clin..

[2] World Health Organization (2024). Breast Cancer Statistics. https://www.who.int/news-room/fact-sheets/detail/breast-cancer.

[3] Łukasiewicz S., Czeczelewski M., Forma A., Baj J., Sitarz R., Stanisławek A. (2021). Breast Cancer-Epidemiology, Risk Factors, Classification, Prognostic Markers, and Current Treatment Strategies-An Updated Review. Cancers.

[4] Shang C., Xu D. (2022). Epidemiology of Breast Cancer. Oncologie.

[5] Taskindoust M., Thomas S.M., Sammons S.L., Fayanju O.M., DiLalla G., Hwang E.S., Plichta J.K. (2021). Survival Outcomes Among Patients with Metastatic Breast Cancer: Review of 47,000 Patients. Ann. Surg. Oncol..

[6] Bergerot C., Bergerot P.G., Maués J., Segarra-Vazquez B., Mano M.S., Tarantino P. (2024). Is Cancer Back? Psychological Issues Faced by Survivors of Breast Cancer. Ann. Palliat. Med..

[7] Secinaro S., Calandra D., Secinaro A., Muthurangu V., Biancone P. (2021). The Role of Artificial Intelligence in Healthcare: A Structured Literature Review. BMC Med. Inform. Decis. Mak..

[8] Panch T., Szolovits P., Atun R. (2018). Artificial Intelligence, Machine Learning and Health Systems. J. Glob. Health.

[9] Kourou K., Exarchos T.P., Exarchos K.P., Karamouzis M.V., Fotiadis D.I. (2015). Machine Learning Applications in Cancer Prognosis and Prediction. Comput. Struct. Biotechnol. J..

[10] Shah S.M., Khan R.A., Arif S., Sajid U. (2022). Artificial Intelligence for Breast Cancer Analysis: Trends and Directions. Comput. Biol. Med..

[11] Khurana D., Koli A., Khatter K., Singh S. (2023). Natural Language Processing: State of the Art, Current Trends and Challenges. Multimed. Tools Appl..

[12] Sarella P.N.K., Mangam V.T. (2024). AI-Driven Natural Language Processing in Healthcare: Transforming Patient-Provider Communication. Indian J. Pharm. Pract..

[13] Wu W.-T., Li Y.-J., Feng A.-Z., Li L., Huang T., Xu A.-D., Lyu J. (2021). Data Mining in Clinical Big Data: The Frequently Used Databases, Steps, and Methodological Models. Mil. Med. Res..

[14] Alpers L.M. (2018). Distrust and Patients in Intercultural Healthcare: A Qualitative Interview Study. Nurs. Ethics.

[15] Chaix B., Bibault J.-E., Pienkowski A., Delamon G., Guillemasse A., Nectoux P., Brouard B. (2019). When Chatbots Meet Patients: One-Year Prospective Study of Conversations Between Patients with Breast Cancer and a Chatbot. JMIR Cancer.

[16] Bayley E.M., Liu H.Y., Bonetti M.A., Egro F.M., Diego E.J. (2024). ChatGPT as Valuable Patient Education Resource in Breast Cancer Care. Ann. Surg. Oncol..

[17] Gummadi R., Dasari N., Kumar S.D., Pindiprolu S.K.S. (2024). Evaluating the Accuracy of Large Language Model (ChatGPT) in Providing Information on Metastatic Breast Cancer. Adv. Pharm. Bull..

[18] Liu Y.H., Bonetti A.M., Jeong T., Pandya S., Nguyen V.T., Egro M.F. (2023). Dr. ChatGPT Will See You Now: How Do Google and ChatGPT Compare in Answering Patient Questions on Breast Reconstruction?. J. Plast. Reconstr. Aesthetic. Surg..

[19] Pan A., Musheyev D., Bockelman D., Loeb S., Kabarriti A.E. (2023). Assessment of Artificial Intelligence Chatbot Responses to Top Searched Queries About Cancer. JAMA Oncol..

[20] Haug C.J., Drazen J.M. (2023). Artificial Intelligence and Machine Learning in Clinical Medicine, 2023. N. Engl. J. Med..

[21] Sidey-Gibbons J.A.M., Sidey-Gibbons C.J. (2019). Machine Learning in Medicine: A Practical Introduction. BMC Med. Res. Methodol..

[22] Pan L., Wu X., Lu Y., Zhang H., Zhou Y., Liu X., Liu S., Yan Q. (2022). Artificial Intelligence Empowered Digital Health Technologies in Cancer Survivorship Care: A Scoping Review. Asia Pac. J. Oncol. Nurs..

[23] Bi W.L., Hosny A., Schabath M.B., Giger M.L., Birkbak N.J., Mehrtash A., Allison T., Arnaout O., Abbosh C., Dunn I.F. (2019). Artificial Intelligence in Cancer Imaging: Clinical Challenges and Applications. CA Cancer J. Clin..

[24] Chia J.L.L., He G.S., Ngiam K.Y., Hartman M., Ng Q.X., Goh S.S.N. (2025). Harnessing Artificial Intelligence to Enhance Global Breast Cancer Care: A Scoping Review of Applications, Outcomes, and Challenges. Cancers.

[25] Peters M.D.J., Marnie C., Colquhoun H., Garritty C.M., Hempel S., Horsley T., Langlois E.V., Lillie E., O’bRien K.K., Tunçalp O. (2021). Scoping Reviews: Reinforcing and Advancing the Methodology and Application. Syst. Rev..

[26] Tricco A.C., Lillie E., Zarin W., O’Brien K.K., Colquhoun H., Levac D., Moher D., Peters M.D.J., Horsley T., Weeks L. (2018). PRISMA Extension for Scoping Reviews (PRISMA-ScR): Checklist and Explanation. Ann. Intern. Med..

[27] Seripenah P., Prudence I., Oni G., Polotto S., Leonardi-Bee J., Morling J., Joseph T., Koboto D., Dhungana S., Emery H. (2023). Use of Artificial Intelligence (AI) in Breast Patient Care: A Scoping Review Protocol. OSF Preprints. https://osf.io/a2vgj.

[28] World Health Organization (2021). Ethics and Governance of Artificial Intelligence for Health.

[29] Topol E.J. (2019). High-Performance Medicine: The Convergence of Human and Artificial Intelligence. Nat. Med..

[30] Perez K., Wisniewski D., Ari A., Lee K., Lieneck C., Ramamonjiarivelo Z. (2025). Investigation into Application of AI and Telemedicine in Rural Communities: A Systematic Literature Review. Healthcare.

[31] Al-Allak A., Intabli L., Bertelli G., Lewis P. (2017). Artificial intelligence: A new generation of intelligent predictive models to guide adjuvant treatment decisions for patients with breast cancer?. Eur. J. Surg. Oncol..

[32] Badwe R., Thaker P., Begum F., Acherjee M., Srivastava G., Ramarajan N., Nag S., Gupta S., Gulia S., Ghosh J. (2024). Abstract PO4-10-10: NAVYA-AI enabled intervention to increase real-world guideline compliant care: Improving NGS testing in breast cancer. Cancer Res..

[33] Bakx N., Bluemink H., Hagelaar E., van Gruijthuijsen D., van der Leer J., van Nunen T., van der Sangen M., Theuws J., Hurkmans C. (2023). Evaluation of the Clinical Use of a Deep Learning Model for Breast Cancer Treatment Plan Generation. Radiother. Oncol..

[34] Banks P., Cavedon L., Verspoor K., Pitson G. (2016). Content Analysis of Clinical Letters for Breast Cancer Patients in the Adjuvant Setting: The First Step Toward Automated Extraction of Clinical Data. Asia Pac. J. Clin. Oncol..

[35] Bayram E., Basaran G., Gokmen E., Mandel N.M., Oyan B., Uskent N., Karagoz B., Yildirim Y., Karaaslan S., Guler E.N. (2024). Artificial Intelligence Algorithms for Recurrence Risk Score Prediction in Early-Stage Breast Cancer: A Multicenter Study of 437 Cases. J. Clin. Oncol..

[36] Chetlen A., Artrip R., Drury B., Arbaiza A., Moore M. (2019). Novel Use of Chatbot Technology to Educate Patients Before Breast Biopsy. J. Am. Coll. Radiol..

[37] D’Onofrio G., D’Amore A., Onofaro F., Caputi E., Napoli A., Triassi M., Marino M.R. (2023). Prediction of Hospital Length of Stay for Patients Undergoing Mastectomy. Stud. Health Technol. Inform..

[38] Fiandra C., Cattani F., Leonardi M.C., Comi S., Zara S., Rossi L., Jereczek-Fossa B., Ricardi U., Heijmen B. (2022). Machine Learning to Predict the Quality of a Left-Sided Whole Breast Radiotherapy Treatment Plan. Int. J. Radiat. Oncol. Biol. Phys..

[39] Ozgür E.G., Ulgen A., Uzun S., Bekiroglu G.N. (2024). Evaluation of Risk Factors and Survival Rates of Patients with Early-Stage Breast Cancer with Machine Learning and Traditional Methods. Int. J. Med. Inform..

[40] Pfob A., Mehrara B., Nelson J., Wilkins E.G., Pusic A., Sidey-Gibbons C. (2020). Towards Data-Driven Decision-Making for Breast Cancer Patients Undergoing Mastectomy and Reconstruction: Prediction of Individual Patient-Reported Outcomes at Two-Year Follow-Up Using Machine Learning. J. Clin. Oncol..

[41] Ren J., Li Y., Zhou J., Yang T., Jing J., Xiao Q., Duan Z., Xiang K., Zhuang Y., Li D. (2024). Developing Machine Learning Models for Personalized Treatment Strategies in Early Breast Cancer Patients Undergoing Neoadjuvant Systemic Therapy Based on SEER Database. Sci. Rep..

[42] Romo-Bucheli D., Janowczyk A., Gilmore H., Romero E., Madabhushi A. (2017). A Deep Learning Based Strategy for Identifying and Associating Mitotic Activity with Gene Expression Derived Risk Categories in Estrogen Receptor Positive Breast Cancers. Cytom. A.

[43] Stathonikos N., Aubreville M., de Vries S., Wilm F., Bertram C.A., Veta M., van Diest P.J. (2024). Breast Cancer Survival Prediction Using an Automated Mitosis Detection Pipeline. J. Pathol. Clin. Res..

[44] Le Thien M.-A., Redjdal A., Bouaud J., Seroussi B. (2021). Deep Learning, a Not so Magical Problem Solver: A Case Study with Predicting the Complexity of Breast Cancer Cases. Stud. Health Technol. Inform..

[45] Wheeler S.B., Spees L., Biddell C.B., Rotter J., Trogdon J.G., Birken S.A., Mayer D. (2020). Development of a Personalized Follow-Up Care Algorithm for Medicare Breast Cancer Survivors. J. Clin. Oncol..

[46] Yang Q., Luo T., Zhang W., Zhong X., He P., Zheng H. (2022). Data-Driven Treatment Pathways Mining for Early Breast Cancer Using cSPADE Algorithm and System Clustering. Int. J. Health Plann. Manag..

[47] Zarean Shahraki S., Azizmohammad Looha M., Mohammadi Kazaj P., Aria M., Akbari A., Emami H., Asadi F., Akbari M.E. (2023). Time-Related Survival Prediction in Molecular Subtypes of Breast Cancer Using Time-to-Event Deep-Learning-Based Models. Front. Oncol..

[48] DeWees T.A., Golafshar M.A., Bhangoo R.S., Thorpe C.S., Vern-Gross T.Z., McGee L.A., Wong W., Halyard M., Keole S., Vargas C. (2020). Artificial Neural Networks Utilizing Standardly Collected Electronic Healthcare Data Provide Clinically Interpretable Predictions of Patient-Reported Adverse Events for Breast Cancer. Int. J. Radiat. Oncol. Biol. Phys..

[49] Hassan M.A., Biaggi P.A., Asaad M., Andejani F.D., Liu J., Offodile A.C., Selber J.C., Butler C.E. (2023). Development and Assessment of Machine Learning Models for Individualized Risk Assessment of Mastectomy Skin Flap Necrosis. Ann. Surg..

[50] Johnson H., Ali A., Zhang X., Wang T., Simoulis A., Wingren A.G., Persson J.L. (2022). K-RAS Associated Gene-Mutation-Based Algorithm for Prediction of Treatment Response of Patients with Subtypes of Breast Cancer and Especially Triple-Negative Cancer. Cancers.

[51] Ma D.C., Potters L., Lee L., Bloom B.F., Andrews J.Z., Chen W., Teckie S. (2021). Patient-Reported Outcomes Using Automated Chatbot for Breast Cancer Patients Receiving Radiation Therapy. Int. J. Radiat. Oncol. Biol. Phys..

[52] Tawfik E., Ghallab E., Moustafa A. (2023). A Nurse versus a Chatbot: The Effect of an Empowerment Program on Chemotherapy-Related Side Effects and the Self-Care Behaviors of Women Living with Breast Cancer: A Randomized Controlled Trial. BMC Nurs..

[53] Banerjee I., Bozkurt S., Caswell-Jin L.J., Kurian A.W., Rubin D.L. (2019). Natural Language Processing Approaches to Detect the Timeline of Metastatic Recurrence of Breast Cancer. JCO Clin. Cancer Inform..

[54] Boeri C., Chiappa C., Galli F., De Berardinis V., Bardelli L., Carcano G., Rovera F. (2020). Machine Learning Techniques in Breast Cancer Prognosis Prediction: A Primary Evaluation. Cancer Med..

[55] Calabrese A., Santucci D., Gravina M., Faiella E., Cordelli E., Soda P., Iannello G., Sansone C., Zobel B.B., Catalano C. (2022). 3T-MRI Artificial Intelligence in Patients with Invasive Breast Cancer to Predict Distant Metastasis Status: A Pilot Study. Cancers.

[56] Chae E.Y., Jung M.R., Cha J.H., Shin H.J., Choi W.J., Kim H.H. (2024). A Predictive Model Using MRI and Clinicopathologic Features for Breast Cancer Recurrence in Young Women Treated with Upfront Surgery. Eur. Radiol..

[57] Deutsch M.T., Pfob A., Brusniak K., Riedel F., Bauer A., Dijkstra T., Engler T., Brucker S.Y., Hartkopf A.D., Schneeweiss A. (2023). Machine Learning and Patient-Reported Outcomes for Longitudinal Monitoring of Disease Progression in Metastatic Breast Cancer: A Multicenter, Retrospective Analysis. Eur. J. Cancer.

[58] Donovan M.J., Meurs C.J.C., Fernandez G., Madduri A.S., Feliz A., Zeineh J., DeAngel R., Westenend P. (2024). AI-Enabled Digital Test to Predict Disease Recurrence for Patients with Early-Stage Invasive Breast Cancer and Performance in a MammaPrint Low-Risk Cohort from the Netherlands with a Median 6-Year Follow-Up. J. Clin. Oncol..

[59] Donovan M.J., Westenend P., Meurs C., Fernandez G., Zeineh J., Madduri A.S., Feliz A., Shtabsky A., Zhang X., Veremis B. (2024). External Validation of an AI-Enabled Digital Test (PreciseDx Breast) to Predict Breast Cancer Recurrence within 6-Years Exhibited Good Independent Performance on a Comparable Early-Stage Cohort from the Netherlands. Ann. Oncol..

[60] Donovan M., Fernandez G., Zeineh J., Madduri S.A., Scott R., Prastawa M. (2024). Clinical Validation of an Artificial-Intelligent (AI) Enabled Digital Test Using the Patient’s Diagnostic Breast Biopsy to Predict Invasive Breast Cancer Recurrence within 6-Years. Eur. J. Cancer.

[61] Du Y., Zhou X., Gao Q., Yang C., Huang T. (2025). A Deep Reinforcement Learning-Based Feature Selection Method for Invasive Disease Event Prediction Using Imbalanced Follow-Up Data. IEEE J. Biomed. Health Inform..

[62] Fernandez G., Prastawa M., Madduri S.A., Scott R., Marami B., Shpalensky N., Cascetta K., Sawyer M., Chan M., Koll G. (2022). Development and Validation of an AI-Enabled Digital Breast Cancer Assay to Predict Early-Stage Breast Cancer Recurrence within 6 Years. Breast Cancer Res..

[63] Gonzalez-Castro L., Chavez M., Duflot P., Bleret V., Martin A.G., Zobel M., Nateqi J., Lin S., Pazos-Arias J.J., Del Fiol G. (2023). Machine Learning Algorithms to Predict Breast Cancer Recurrence Using Structured and Unstructured Sources from Electronic Health Records. Cancers.

[64] Izci H., Macq G., Tambuyzer T., De Schutter H., Wildiers H., Duhoux F.P., de Azambuja E., Taylor D., Staelens G., Orye G. (2023). Machine Learning Algorithm to Estimate Distant Breast Cancer Recurrence at the Population Level with Administrative Data. Clin. Epidemiol..

[65] Kim J.-Y., Lee S.Y., Yu J., Park Y., Lee K.S., Lee M., Lee J.E., Kim S.W., Nam S.J., Park Y.H. (2021). Deep Learning-Based Prediction Model for Breast Cancer Recurrence Using Adjuvant Breast Cancer Cohort in Tertiary Cancer Center Registry. Front. Oncol..

[66] Lötsch J., Ultsch A., Kalso E. (2017). Prediction of Persistent Post-Surgery Pain by Preoperative Cold Pain Sensitivity: Biomarker Development with Machine-Learning-Derived Analysis. Br. J. Anaesth..

[67] Lou S.-J., Hou M.-F., Chang H.-T., Chiu C.-C., Lee H.-H., Yeh J.S.C., Shi H.-Y. (2020). Machine Learning Algorithms to Predict Recurrence within 10 Years after Breast Cancer Surgery: A Prospective Cohort Study. Cancers.

[68] Moreau N., Rousseau C., Fourcade C., Santini G., Ferrer L., Lacombe M., Guillerminet C., Campone M., Colombie M., Rubeaux M. (2020). Deep Learning Approaches for Bone and Bone Lesion Segmentation on 18FDG PET/CT Imaging in the Context of Metastatic Breast Cancer. Annu. Int. Conf. IEEE Eng. Med. Biol. Soc..

[69] Murata T., Yoshida M., Shiino S., Ogawa A., Watase C., Satomi K., Jimbo K., Maeshima A., Iwamoto E., Takayama S. (2023). A Prediction Model for Distant Metastasis after Isolated Locoregional Recurrence of Breast Cancer. Breast Cancer Res. Treat..

[70] Prastawa M., Madduri S.A., Veremis B., Shtabsky A., Marami B., Zeineh J., Donovan M.J., Fernandez G. (2020). The Application of Machine Learning Techniques to Standardize Breast Cancer Grading and Develop Multivariate Risk Outcome Models. Cancer Res..

[71] Rong R.Y., Shen Y.K., Wu S.N., Xu S.H., Hu J.Y., Zou J., He L., Chen C., Kang M., Ying P. (2024). Prediction Model for Ocular Metastasis of Breast Cancer: Machine Learning Model Development and Interpretation Study. BMC Cancer.

[72] Shin D.S., Lee J., Cheun J.H., Lee J.H., Shin Y., Bae J.S., Kang E., Kwon S., Lee H.-B., Ryu J.M. (2024). Machine Learning-Based Risk Prediction for Late Distant Recurrence in Young Women with Estrogen Receptor-Positive/Human Epidermal Growth Factor 2-Negative Breast Cancer. Cancer Res..

[73] Syleouni M.-E., Karavasiloglou N., Manduchi L., Wanner M., Korol D., Rohrmann S. (2023). Predicting Second Breast Cancer among Women with Primary Breast Cancer Using Machine Learning Algorithms, a Population-Based Observational Study. Int. J. Cancer.

[74] Tseng Y.-J., Huang C.-E., Wen C.-N., Lai P.-Y., Wu M.-H., Sun Y.-C., Wang H.-Y., Lu J.-J. (2019). Predicting Breast Cancer Metastasis by Using Serum Biomarkers and Clinicopathological Data with Machine Learning Technologies. Int. J. Med. Inform..

[75] Vaidya V.P., Agrawal S., Nagdewani S., Chandrashekaraiah P., Bhardwaj T., Narayanan B. (2020). Development of an Artificial Intelligence Model to Dynamically Predict Metastatic Recurrence of Early-Stage Breast Cancer Patients. J. Clin. Oncol..

[76] Varma G., Yenukoti R.K., Kumar M.P., Ashrit B.S., Purushotham K., Subash C., Ravi S.K., Kurien V., Aman A., Manoharan M. (2024). A Deep Learning-Enabled Workflow to Estimate Real-World Progression-Free Survival in Patients with Metastatic Breast Cancer. J. Clin. Oncol..

[77] Zeng L., Liu L., Chen D., Lu H., Xue Y., Bi H., Yang W. (2023). The Innovative Model Based on Artificial Intelligence Algorithms to Predict Recurrence Risk of Patients with Postoperative Breast Cancer. Front. Oncol..

[78] Zhen M., Chen H., Lu Q., Li H., Yan H., Wang L. (2024). Machine Learning-Based Predictive Model for Mortality in Female Breast Cancer Patients Considering Lifestyle Factors. Cancer Manag. Res..

[79] Oh S., Shim J.Y. (2024). Development and Validation of a Deep Learning-Based Cardiovascular Disease Risk Prediction Model for Long-Term Breast Cancer Survivors. J. Clin. Oncol..

[80] Pfob A., Mehrara J.B., Nelson A.J., Wilkins E.G., Pusic A.L., Sidey-Gibbons C. (2021). Machine Learning to Predict Individual Patient-Reported Outcomes at 2-Year Follow-Up for Women Undergoing Cancer-Related Mastectomy and Breast Reconstruction (INSPiRED-001). Breast.

[81] Senthilmahesh H., Nagappan M.P., Shekhar S.N.C., Pandian A.A., Kumar P.S.V.V.S.R., Tumaati R. (2025). Advancing Oncology Care with AI-Powered Virtual Assistants and Chatbots: A Qualitative Exploration of Future Potential and Challenges. Eurasian J. Med. Oncol..

[82] Lawson McLean A., Hristidis V. (2025). Evidence-Based Analysis of AI Chatbots in Oncology Patient Education: Implications for Trust, Perceived Realness, and Misinformation Management. J. Cancer Educ..

[83] Roustan D., Bastardot F. (2025). The Clinicians’ Guide to Large Language Models: A General Perspective with a Focus on Hallucinations. Interact. J. Med. Res..

[84] Chen S., Kann B.H., Foote M.B., Aerts H.J.W.L., Savova G.K., Mak R.H., Bitterman D.S. (2023). Use of Artificial Intelligence Chatbots for Cancer Treatment Information. JAMA Oncol..

[85] Rajkomar A., Hardt M., Howell M.D., Corrado G., Chin M.H. (2018). Ensuring Fairness in Machine Learning to Advance Health Equity. Ann. Intern. Med..

[86] Gianfrancesco M.A., Tamang S., Yazdany J., Schmajuk G. (2018). Potential Biases in Machine Learning Algorithms Using Electronic Health Record Data. JAMA Intern. Med..

[87] Akhtar Z.B. (2024). Unveiling the Evolution of Generative AI (GAI): A Comprehensive and Investigative Analysis toward LLM Models (2021–2024) and Beyond. J. Electr. Syst. Inf. Technol..

[88] Wang B., Asan O., Mansouri M. (2023). What May Impact Trustworthiness of AI in Digital Healthcare: Discussion from Patients’ Viewpoint. Proc. Int. Symp. Hum. Factors Ergon. Health Care.

[89] Khullar D., Casalino L.P., Qian Y., Lu Y., Krumholz H.M., Aneja S. (2022). Perspectives of Patients About Artificial Intelligence in Health Care. JAMA Netw. Open.

