# Supplementary material for: Applications of Artificial Intelligence (AI) in Breast Cancer Care Delivery and Education: A Scoping Review
Source: Int J Environ Res Public Health. 2026 Apr 23;23(5):545. doi: 10.3390/ijerph23050545 (PMC13206135; doi:10.3390/ijerph23050545)
Supplement: Supplementary file 1 [file ijerph-23-00545-s001.zip › File S1. Full electronic search strategies for each database.pdf]

# Supplementary Material File S1: Full electronic search strategies presented exactly as run in each database

*Table S1. MEDLINE via OVID*

Search Conducted: December 10th, 2024

Records Retrieved: 712

| Search # | Query | Results |
|---|---|---|
| 1 | ("Artificial* intellig*" or "machine learning" or "deep learning" or "predictive model*" or algorithm* or "chatbot*" or "algorithms" or "supervised machine learning" or "unsupervised machine learning" or "computer-aided").ti,ab. | 602751 |
| 2 | Artificial Intelligence/ | 51106 |
| 3 | ("AI" or "Artificial Neural Network" or "neural network*" or "Neural Networks").tw. | 175293 |
| 4 | ("breast cancer" or "breast neoplasm" or "Breast Neoplasms" or "Breast Tumor" or "Breast Carcinoma").ti,ab. | 380602 |
| 5 | Breast Cancer.mp. or Breast Neoplasms/ | 473760 |
| 6 | ("Patient Education" or "Patient Management" or "Patient Support" or "Healthcare Management" or "treatment plan" or "follow-up").tw. | 1376391 |
| 7 | Patient Education.mp. or Patient Education as Topic/ | 112854 |
| 8 | 1 or 2 or 3 | 708284 |
| 9 | 4 or 5 | 480148 |
| 10 | 6 or 7 | 1458080 |
| 11 | 8 and 9 and 10 | 1010 |
| 12 | (8 and 9 and 10) not ("Breast Cancer Screening" or "Breast Cancer Detection").mp. | 948 |
| 13 | limit 12 to yr="2013 - 2024" | 712 |

*Table S2. EMBASE via OVID*

Search Conducted: December 10th, 2024

Records Retrieved: 1741

| Search # | Query | Results |
| --- | --- | --- |
| 1 | ("Artificial* intellig*" or "machine learning" or "deep learning" or "predictive model*" or algorithm* or "chatbot*" or "algorithms" or "supervised machine learning" or "unsupervised machine learning" or "computer-aided").ti,ab. | 741409 |
| 2 | Artificial Intelligence/ | 92121 |
| 3 | ("AI" or "Artificial Neural Network" or "neural network*" or "Neural Networks").tw. | 212548 |
| 4 | ("breast cancer" or "breast neoplasm" or "Breast Neoplasms" or "Breast Tumor" or "Breast Carcinoma").ti,ab. | 552940 |
| 5 | Breast Cancer.mp. or Breast Neoplasms/ | 699389 |
| 6 | ("Patient Education" or "Patient Management" or "Patient Support" or "Healthcare Management" or "treatment plan" or "follow-up").tw. | 2209080 |
| 7 | Patient Education.mp. or Patient Education as Topic/ | 142957 |
| 8 | 1 or 2 or 3 | 880469 |
| 9 | 4 or 5 | 718208 |
| 10 | 6 or 7 | 2307263 |
| 11 | 8 and 9 and 10 | 2277 |
| 12 | (8 and 9 and 10) not ("Breast Cancer Screening" or "Breast Cancer Detection").mp. | 2186 |
| 13 | limit 12 to yr="2013 - 2024" | 1741 |

*Table S3. CINAHL*

Search Conducted: December 10th, 2024

Records Retrieved: 63

| Search # | Query | Limiters/Expanders | Results |
|---|---|---|---|
| S1 | (MH "Deep Learning") OR (MH "Machine Learning") OR<br>""Artificial* intellig*" OR "machine learning" OR "deep<br>learning" OR "predictive model*" OR algorithm* OR<br>"chatbot*" OR "supervised machine learning" OR<br>"unsupervised machine learning" OR "computer-aided"" | Limiters - Exclude MEDLINE records<br><br>Expanders - Apply equivalent subjects<br><br>Search modes - Proximity | 9943 |
| S2 | MH "Artificial Intelligence" | Limiters - Exclude MEDLINE records<br><br>Expanders - Apply equivalent subjects<br><br>Search modes - Proximity | 11120 |
| S3 | "AI" OR "Artificial Neural Network" OR "neural network*" | Limiters - Exclude MEDLINE records<br><br>Expanders - Apply equivalent subjects<br><br>Search modes - Proximity | 17119 |

| | | | |
|---|---|---|---|
| S4 | (MH "Breast Neoplasms") OR "breast cancer OR breast neoplasm OR Breast Neoplasms OR Breast Tumor OR Breast Carcinoma" | Limiters - Exclude MEDLINE records<br><br>Expanders - Apply equivalent subjects<br><br>Search modes - Proximity | 44190 |
| S5 | (MM "Breast Care") | Limiters - Exclude MEDLINE records<br><br>Expanders - Apply equivalent subjects<br><br>Search modes - Proximity | 110 |
| S6 | Patient Education OR Patient Management OR Patient Support OR healthcare management OR treatment plan OR follow-up | Limiters - Exclude MEDLINE records<br><br>Expanders - Apply equivalent subjects<br><br>Search modes - Proximity | 226440 |
| S7 | (MH "Patient Education") | Limiters - Exclude MEDLINE records<br><br>Expanders - Apply equivalent subjects | 41358 |

| | | | |
|---|---|---|---|
| | | Search modes - Proximity | |
| S8 | S1 or S2 or S3 | Expanders - Apply equivalent subjects Search modes - Proximity | 26587 |
| S9 | S5 OR S6 OR S7 | Expanders - Apply equivalent subjects Search modes - Proximity | 226545 |
| S10 | S4 AND S8 AND S9 | Expanders - Apply equivalent subjects Search modes - Proximity | 74 |
| S11 | (S4 AND S8 AND S9) NOT ("Breast Cancer Screening" OR "Breast Cancer Detection") | Limiters - Publication Date: 20130101-20241231 Expanders - Apply equivalent subjects Search modes - Proximity | 63 |

*Table S4. Web of Science*

Search Conducted: December 10th, 2024

Records Retrieved: 1268

| Search # | Query | Results |
|---|---|---|
| 1 | AB = ("Artificial* intellig*" or "machine learning" or "deep learning" or "predictive model*" or algorithm* or "chatbot*" or "algorithms" or "supervised machine learning" or "unsupervised machine learning" or "computer-aided") | 3091444 |
| 2 | ALL =(Artificial Intelligence) | 589596 |
| 3 | ALL=("AI" or "Artificial Neural Network" or "neural network*" or "Neural Networks") | 1496998 |
| 4 | #3 OR #2 OR #1 | 4348321 |
| 5 | ALL=("breast cancer" or "breast neoplasm" or "Breast Neoplasms" or "Breast Tumor" or "Breast Carcinoma") | 697045 |
| 6 | ALL=("Patient Education" or "Patient Management" or "Patient Support" or "Healthcare Management" or "treatment plan" or "follow-up" ) | 1519091 |
| 7 | #4 AND #5 AND #6 | 1687 |
| 8 | #4 AND #5 AND #6<br>Timespan: 2013-01-01 to 2024-12-10 | 1268 |
