# Supplementary material for: Applications of Artificial Intelligence (AI) in Breast Cancer Care Delivery and Education: A Scoping Review
Source: Int J Environ Res Public Health. 2026 Apr 23;23(5):545. doi: 10.3390/ijerph23050545 (PMC13206135; doi:10.3390/ijerph23050545)
Supplement: Supplementary file 1 [file ijerph-23-00545-s001.zip › File S2. Blank Data Extraction Instrument.pdf]

Supplementary Material File S2: Blank Data extraction instrument

Author	Year	Country of Study	Article Type	Study Type	Study Population	Study Purpose	Type of AI Identified	Role of AI in Breast Cancer	Post-diagnosis stage of breast cancer
