# Supplementary material for: Applications of Artificial Intelligence (AI) in Breast Cancer Care Delivery and Education: A Scoping Review
Source: Int J Environ Res Public Health. 2026 Apr 23;23(5):545. doi: 10.3390/ijerph23050545 (PMC13206135; doi:10.3390/ijerph23050545)
Supplement: Supplementary file 1 [file ijerph-23-00545-s001.zip › File S3. Summary of included studies.pdf]

Supplementary Material File S3: Summary table of data extracted from included studies

Table S1. Summary table of data extracted from included studies (n=54).

**Lead Author, Year, Country**	**Article Type**	**Study Type**	**Study Purpose**	**Type of AI Identified**	**AI Role**	**AI Population Focus**	**Post-Diagnosis Care Stage**
Al-Allak, 2017, United Kingdom [31]	Conference Abstract	Retrospective Cohort	Develop prognostic tool to predict survival and guide adjuvant decisions.	Machine Learning	Survival Prediction	Provider-Focused	Treatment Planning
Badwe, 2024, India [32]	Conference Abstract	Retrospective Cohort	Assess physician compliance with National Comprehensive Cancer Network testing guidelines via Navya-AI.	Machine Learning	Workflow Optimization	Provider-Focused	Treatment Planning
Bakx, 2023, Netherlands [33]	Conference Abstract	Cross-sectional Study	Evaluate AI for dose prediction and automated radiotherapy treatment planning.	Machine Learning	Treatment Planning	Provider-Focused	Treatment Planning

Banks, 2016, Australia [34]	Conference Abstract	Content Analysis	Content analysis of clinical letters to extract treatment patterns.	Machine Learning	Workflow Optimization	Provider-Focused	Treatment Planning
Bayram, 2024, Turkey [35]	Conference Abstract	Retrospective Cohort	Predict recurrence risk score to assist early-stage adjuvant decisions.	Machine Learning	Recurrence Prediction	Provider-Focused	Treatment Planning
Chetlen, 2019, United States [36]	Journal Article	Pilot Study	Use chatbot to educate patients on what to expect before a breast biopsy.	Conversational Agents (non-LLM)	Patient Support and Education	Patient-Focused	Treatment Planning
D'Onofrio, 2023, Italy [37]	Journal Article	Retrospective Cohort	Predict hospital length of stay for patients undergoing mastectomy.	Machine Learning	Workflow Optimization	Provider-Focused	Treatment Planning
Fiandra, 2022, Italy [38]	Journal Article	Retrospective Cohort	Predict quality of whole breast radiotherapy treatment plans.	Machine Learning	Treatment Planning	Provider-Focused	Treatment Planning
Ozgur, 2024, Turkey [39]	Journal Article	Retrospective Cohort	To compare conventional statistical methods with machine learning techniques for predicting post-treatment survival	Machine Learning	Survival Prediction	Provider-Focused	Treatment Planning

			in early-stage breast cancer.				
Pfob, 2020, North America [40]	Conference Abstract	Retrospective Cohort	Predict individual patient-reported outcomes to guide mastectomy decisions.	Machine Learning	Patient Support and Education	Patient-Focused	Treatment Planning
Ren, 2024, United Kingdom [41]	Journal Article	Retrospective Cohort	Develop models for personalized strategies in neoadjuvant systemic therapy.	Machine Learning	Survival Prediction	Provider-Focused	Treatment Planning
Romo-Bucheli, 2017, Colombia [42]	Journal Article	Retrospective Cohort	Associate mitotic activity with gene-expression risk categories (Oncotype).	Machine Learning	Disease Progression Risk Prediction	Provider-Focused	Treatment Planning
Stathonikos, 2024, Netherlands [43]	Journal Article	Retrospective Cohort	Use automated mitosis detection to predict breast cancer survival.	Machine Learning	Survival Prediction	Provider-Focused	Treatment Planning
Thien, 2021, France [44]	Journal Article	Case Study	Predict complexity of cases to support	Machine Learning	Disease Progression	Provider-Focused	Treatment Planning

			Multidisciplinary Tumour Boards.		Risk Prediction		
Wheeler, 2020, United States [45]	Conference Abstract	Retrospective Cohort	Personalized algorithm to triage survivors to primary vs. specialist care.	Machine Learning	Treatment Planning	Provider-Focused	Treatment Planning
Yang, 2022, China [46]	Journal Article	Retrospective Cohort	Analyse treatment patterns to improve planning efficiency.	Data Mining	Workflow Optimization	Provider-Focused	Treatment Planning
Zarean, 2023, Iran [47]	Journal Article	Retrospective Cohort	Predict long-term survival probabilities across molecular subtypes.	Machine Learning	Survival Prediction	Provider-Focused	Treatment Planning
DeWees, 2020, United States [48]	Conference Abstract	Longitudinal Study	Predict patient-reported adverse events using EHR data.	Machine Learning	Adverse Event Prediction	Provider-Focused	Treatment Delivery
Hassan, 2023, United States [49]	Journal Article	Retrospective Cohort	Risk assessment of mastectomy skin flap necrosis during surgery.	Machine Learning	Adverse Event Prediction	Provider-Focused	Treatment Delivery

Johnson, 2022, United States [50]	Journal Article	Retrospective Cohort	Gene-mutation algorithm to predict response to triple-negative treatment.	Machine Learning	Treatment Response Prediction	Provider-Focused	Treatment Delivery
Ma, 2021, United States [51]	Conference Abstract	Cross-sectional Study	Chatbot for patients receiving radiation therapy to track outcomes.	Conversational Agents (non-LLM)	Patient Support and Education	Patient-Focused	Treatment Delivery
Tawfik, 2023, Egypt [52]	Journal Article	Randomized Control Trial	Empower women to manage chemotherapy side effects via a chatbot.	Conversational Agents (non-LLM)	Patient Support and Education	Patient-Focused	Treatment Delivery
Banerjee, 2019, United States [53]	Journal Article	Retrospective Cohort	NLP algorithm to detect metastatic recurrence from clinical text notes.	Traditional Natural Language Processing (tNLP)	Recurrence Prediction	Provider-Focused	Follow-up & Surveillance
Boeri, 2020, Italy [54]	Journal Article	Retrospective Cohort	Primary evaluation of ML for prognosis and recurrence prediction.	Machine Learning	Recurrence Prediction	Provider-Focused	Follow-up & Surveillance
Calabrese, 2022, Italy [55]	Journal Article	Retrospective Cohort	MRI-based AI to predict distant metastasis status.	Machine Learning	Recurrence Prediction	Provider-Focused	Follow-up & Surveillance

Chae, 2024, South Korea [56]	Journal Article	Retrospective Cohort	Predict recurrence in young women using MRI and clinicopathologic data.	Machine Learning	Recurrence Prediction	Provider-Focused	Follow-up & Surveillance
Deutsch, 2023, Germany [57]	Journal Article	Longitudinal Study	Longitudinal monitoring of disease progression in metastatic cases.	Machine Learning	Disease Progression Risk Prediction	Provider-Focused	Follow-up & Surveillance
Donovan, 2024, Netherlands [58]	Conference Abstract	Retrospective Cohort	AI-enabled digital test to predict BCR recurrence within 6 years.	Machine Learning	Recurrence Prediction	Provider-Focused	Follow-up & Surveillance
Donovan, 2024, Netherlands [59]	Conference Abstract	Retrospective Cohort	External validation of PDxBr AI-enabled digital test for recurrence prediction within 6 years.	Machine Learning	Recurrence Prediction	Provider-Focused	Follow-up & Surveillance
Donovan, 2024, United States [60]	Journal Article	Retrospective Cohort	Clinical validation of AI-enabled digital test using diagnostic biopsy for recurrence prediction within 6 years.	Machine Learning	Recurrence Prediction	Provider-Focused	Follow-up & Surveillance

Du, 2024, China [61]	Journal Article	Cross-sectional Study	Deep reinforcement learning for invasive disease event prediction.	Machine Learning	Disease Progression Risk Prediction	Provider-Focused	Follow-up & Surveillance
Fernandez, 2022, United States [62]	Journal Article	Retrospective Cohort	Digital breast cancer assay to predict early-stage recurrence.	Machine Learning	Recurrence Prediction	Provider-Focused	Follow-up & Surveillance
Gonzalez-Castro, 2023, Belgium [63]	Journal Article	Retrospective Cohort	Predict recurrence using structured/unstructured sources from EHRs.	Machine Learning	Recurrence Prediction	Provider-Focused	Follow-up & Surveillance
Izci, 2022, Belgium [64]	Journal Article	Retrospective Cohort	Population-level algorithm to estimate distant recurrence.	Machine Learning	Recurrence Prediction	Provider-Focused	Follow-up & Surveillance
Kim, 2021, South Korea [65]	Journal Article	Retrospective Cohort	Predict recurrence using tertiary cancer center registry data.	Machine Learning	Recurrence Prediction	Provider-Focused	Follow-up & Surveillance
Lötsch, 2017, Finland [66]	Journal Article	Retrospective Cohort	Predict persistent post-surgery pain using cold pain sensitivity data.	Machine Learning	Adverse Event Prediction	Provider-Focused	Follow-up & Surveillance

Lou, 2020, Taiwan [67]	Journal Article	Longitudinal Study	Predict recurrence within 10 years after breast cancer surgery.	Machine Learning	Recurrence Prediction	Provider-Focused	Follow-up & Surveillance
Moreau, 2020, France [68]	Journal Article	Prospective Cohort	Deep Learning for bone lesion segmentation in metastatic care.	Machine Learning	Disease Progression Risk Prediction	Provider-Focused	Follow-up & Surveillance
Murata, 2023, Japan [69]	Journal Article	Retrospective Cohort	Predict distant metastasis after isolated locoregional recurrence.	Machine Learning	Recurrence Prediction	Provider-Focused	Follow-up & Surveillance
Prastawa, 2020, United States [70]	Conference Abstract	Retrospective Cohort	Application of machine learning to standardize grading and develop risk models.	Machine Learning	Survival Prediction	Provider-Focused	Follow-up & Surveillance
Rong, 2024, China [71]	Journal Article	Retrospective Cohort	Predictive model for ocular metastasis in breast cancer patients.	Machine Learning	Disease Progression Risk Prediction	Provider-Focused	Follow-up & Surveillance
Shin, 2024, Netherlands [72]	Conference Abstract	Retrospective Cohort	Risk prediction for late distant recurrence in	Machine Learning	Recurrence Prediction	Provider-Focused	Follow-up & Surveillance

			young women (ER+/HER2-).				
Syleouni, 2023, Switzerland [73]	Journal Article	Retrospective Cohort	Predict second breast cancers among women with primary diagnosis.	Machine Learning	Recurrence Prediction	Provider-Focused	Follow-up & Surveillance
Tseng, 2019, Taiwan [74]	Journal Article	Retrospective Cohort	Predict metastasis using serum biomarkers and clinicopathologic data.	Machine Learning	Recurrence Prediction	Provider-Focused	Follow-up & Surveillance
Vaidya, 2020, Not Stated [75]	Conference Abstract	Retrospective Cohort	Dynamic prediction of metastatic recurrence in early-stage patients.	Machine Learning	Recurrence Prediction	Provider-Focused	Follow-up & Surveillance
Varma, 2024, India [76]	Conference Abstract	Retrospective Cohort	To develop and validate a semi-automated workflow combining NLP and structured electronic health record EHR data to evaluate real-world progression-free survival in metastatic breast cancer patients.	Traditional Natural Language Processing (tNLP)	Disease Progression Risk Prediction	Provider-Focused	Follow-up & Surveillance

Zeng, 2023, China [77]	Journal Article	Retrospective Cohort	Predict recurrence risk from unstructured EHR clinicopathological data.	Machine Learning	Recurrence Prediction	Provider-Focused	Follow-up & Surveillance
Zhen, 2024, China [78]	Journal Article	Retrospective Cohort	Predict mortality integrating clinical and lifestyle factors (SHAP).	Machine Learning	Survival Prediction	Provider-Focused	Follow-up & Surveillance
Bayley, 2024, United States [16]	Journal Article	Cross-sectional Study	Evaluate ChatGPT as a resource for patient education in survivorship.	Generative AI	Patient Support and Education	Patient-Focused	Survivorship Care
Gummadi, 2024, India [17]	Journal Article	Cross-sectional Study	Accuracy of ChatGPT in providing information on metastatic care.	Generative AI	Patient Support and Education	Patient-Focused	Survivorship Care
Liu, 2023, United States [18]	Journal Article	Comparative Observational Study	Compare Google and ChatGPT for answering patient questions.	Generative AI	Patient Support and Education	Patient-Focused	Survivorship Care
Oh, 2024, South Korea [79]	Conference Abstract	Retrospective Cohort	Deep learning-based cardiovascular disease risk in long-term survivors.	Machine Learning	Adverse Event Prediction	Provider-Focused	Survivorship Care

Pan, 2023, United States [19]	Journal Article	Cross-sectional Study	Assessment of four chatbot responses to top searched cancer queries.	Generative AI	Patient Support and Education	Patient-Focused	Survivorship Care
Pfob, 2021, United States, Canada [80]	Journal Article	Longitudinal Study	Predict outcomes (satisfaction) at 2-year follow-up after mastectomy.	Machine Learning	Patient Support and Education	Patient-Focused	Survivorship Care
